# Corrigendum: Clinical manifestations of Kaposi sarcoma herpesvirus lytic activation: multicentric Castleman disease (KSHV–MCD) and the KSHV inflammatory cytokine syndrome

**DOI:** 10.3389/fmicb.2017.01572

**Published:** 2017-08-21

**Authors:** Mark N. Polizzotto, Thomas S. Uldrick, Duosha Hu, Robert Yarchoan

**Affiliations:** HIV/AIDS Malignancy Branch, Center for Cancer Research, National Cancer Institute Bethesda, MD, United States

**Keywords:** Kaposi sarcoma-associated herpesvirus, human herpesvirus 8, multicentric Castleman disease, KSHV Inflammatory cytokine syndrome, interleukin-6, human immunodeficiency virus

In the original article, there was a mistake in Table 2 as published. The units for C reactive protein, ≥3 g/dL, were incorrect, and in fact, because different laboratories have different normal ranges for C reactive protein, we are not providing a specific value. The corrected Table 2 appears below.

**Table 2:** Proposed working case definition of KSHV inflammatory cytokine syndrome.

1. CLINICAL MANIFESTATIONS^*^	
**a. Symptoms**	**b. Laboratory abnormalities**
Fever	Anemia
Fatigue	Thrombocytopenia
Edema	Hypoalbuminemia
Cachexia	Hyponatremia
Respiratory symptoms (including cough, dyspnea, airway hyperreactivity)	**c. Radiographic abnormalities**
Gastrointestinal disturbance (including nausea, anorexia, abdominal discomfort, altered bowel habit)	Lymphadenopathy
Athralgia and myalgia	Splenomegaly
Altered mental state	Hepatomegaly
Neuropathy with or without pain	Body cavity effusions
**2. EVIDENCE OF SYSTEMIC INFLAMMATION**	
Elevated C-reactive protein	
**3. EVIDENCE OF KSHV LYTIC ACTIVITY**	
Elevated KSHV viral load in peripheral blood mononuclear cells (≥100 copies/10^6^ cells)	
**4. NO EVIDENCE OF KSHV-ASSOCIATED MULTICENTRIC cASTLEMAN DISEASE**	
Exclusion of MCD requires pathologic assessment lymph node, bone marrow, or spleen	

*The working case definition of KICS requires the presence of at least two clinical manifestations drawn from at least two categories (1a, b, and c), together with each of the criteria in 2, 3, and 4. Clinical manifestations for the working definition are drawn from the initial case series and from findings commonly seen in KSHV-MCD*.

In addition, the copyright should be considered inaccurate and has been removed to reflect the policies of the United States Government. The authors apologize for this error and state that this does not change the scientific conclusions of the article in any way.

The original article has been updated.

## Conflict of interest statement

The spouse of one of the authors has patents on an assay to measure KSHV vIL-6. These inventions were made as a full-time employee of the US government under 45 Code of Federal Regulations Part 7. All rights, title, and interest to these patents have been assigned to the U.S. Department of Health and Human Services. The government conveys a portion of the royalties it receives to its employee inventors under the Federal Technology Transfer Act of 1986 (P.L. 99–502).

